# Imported Crimean-Congo hemorrhagic fever cases in Istanbul

**DOI:** 10.1186/1471-2334-7-54

**Published:** 2007-06-06

**Authors:** Kenan Midilli, Ayşen Gargılı, Onder Ergonul, Gönül Şengöz, Recep Ozturk, Mehmet Bakar, Frans Jongejan

**Affiliations:** 1 Istanbul University, Cerrahpaşa Medical Faculty, Microbiology and Clinical Microbiology Department, Istanbul; 2 Marmara University, School of Medicine, Infectious Diseases and Clinical Microbiology Department, Istanbul; 3 Ministry of Health of Turkey, Istanbul Unit; 4 Istanbul University, Cerrahpaşa Medical Faculty, Infectious Diseases and Clinical Microbiology Department, Istanbul; 5 Utrecht Centre for Tick-borne Diseases (UCTD), Department of Infectious Diseases and Immunology, Faculty of Veterinary Medicine, Utrecht University, the Netherlands; 6 Department of Veterinary Tropical Diseases, Faculty of Veterinary Science, University of Pretoria, South Africa

## Abstract

We described a series of imported cases of Crimean-Congo Hemorrhagic Fever (CCHF) in Istanbul and investigated the genetic diversity of the virus. All the suspected cases of CCHF, who were applied to the health centers in Istanbul, were screened for CCHF virus (CCHFv) infection by using semi-nested Polymerase Chain Reaction (PCR) following RT-PCR. Simultaneous blood samples were also sent to the national reference laboratory in Ankara for serologic investigation. In 10 out of 91 patients, CCHFv was detected by PCR, and among 9 out of 10, anti-CCHFv IgM antibodies were also positive. Clinical features were characterized by fever, myalgia, and hemorrhage. The levels of liver enzymes, creatinine phosphokinase, and lactate dehydrogenase were elevated, and bleeding markers were prolonged. All the cases were treated with ribavirin. There was no fatal case. All the strains clustered within the same group as other Europe/Turkey isolates.

## Background

Crimean-Congo hemorrhagic fever (CCHF) is a fatal viral infection described in about 30 countries over the world. It has the most extensive geographic range among the medically significant tick-borne viruses [1]. The occurrence of CCHF closely approximates the known world distribution of *Hyalomma *spp. ticks. The virus belongs to the genus *Nairovirus *in the Bunyaviridae family and causes severe diseases in humans, with a reported case fatality rate of 3–30% [1]. CCHF outbreak in Turkey was reported in 2003 [2-4]. By the year 2006, 1103 CCHF confirmed patients were recorded at the Ministry of Health (MOH) of Turkey [5]. The diagnoses of these cases were performed by detection of the virus by PCR or IgM and/or IgG positivity by ELISA. All these patients were hospitalized, 59 of them died, and the case fatality rate was calculated as 5.3%. A great majority of the cases were seen in the eastern Anatolia. No local case was reported from Istanbul yet. However, cases were recently observed in other regions of the country, and the rate of the domestic travel and mobility is high in the country.

Humans become infected through the bites of ticks, by contact with a patient with CCHF during the acute phase of infection, or by contact with blood or tissues from viremic livestock [6]. In 1969, the antigenic structures of the viruses from various geographic regions were reported to be indistinguishable [7]. However, the development of nucleic acid sequence analysis revealed extensive genetic diversity. Furthermore, more data on the genetic sub-types of the viral strains could shed light on the transmission dynamics of the virus. In this study we describe the genetic variation and clinical characteristics of the CCHF cases observed in Istanbul, in the western part of the country.

## Methods

### The Management of the cases

**Figure 1:**
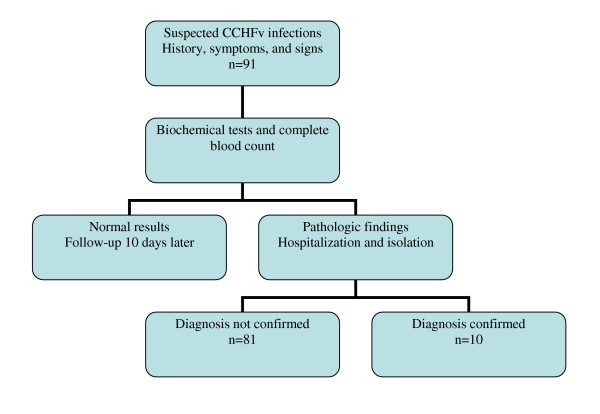
The organization scheme for the suspected cases.

Istanbul unit of the MOH established an *ad hoc *committee for the management of a potential CCHF infection in the city. The group defined the organizational flow for the management of the suspected cases. The suspected cases were defined as the cases with symptoms or signs of CCHF such as fever, myalgia, malaise, and bleeding, and also the history of travel to the endemic region or tick bite. The patients with acute febrile syndrome characterized by malaise, bleeding, leukopenia, and thrombocytopenia in the summer months of 2006 were admitted to the various hospitals of Istanbul (Figure 1).

The patients who had positive IgM and/or IgG and/or positive PCR results for CCHFV in blood were included to the study. The IgM and IgG positivity was detected by using ELISA [8]. Serology for Lyme disease, Salmonella, Leptospira, Rickettsiae, Brucella, and Coxiella infections were investigated. STATA 9.0 (USA) software package was used in the analysis.

### RNA extraction, PCR procedures and construction of phylogenetic tree

Whole blood samples drawn into EDTA containing vacutainer tubes from these suspected cases were sent to Istanbul University Cerrahpaşa Medical Faculty, Microbiology and Clinical Microbiology Department as local reference laboratory. Upon receipt RNA was extracted from 200 μl whole blood using a commercial RNA extraction kit (High Pure Viral Nucleic Acid Kit, Roche Diagnostics^®^, Germany). The extracted RNA and remaining blood were aliquoted and stored at -80°C until work-up. At the admission and the release of the patients blood samples were also sent to the national reference laboratory for serological investigation. Omniscript reverse transcription kit (Qiagen^®^, Germany) was used for the cDNA synthesis in accordance with the instructions of the manufacturer.

Primers from S segment of the virus were selected for the first and second round amplifications. For this purpose, S segment sequences from different geographic regions. (GeneBank accession numbers and origines of the strains: AF362080, China; AF527810, Pakistan; AF481802, Uzbekistan; AJ010648, China; AJ538198, Pakistan; AJ538196, Iraq; AY277672, Russia; AY277676, Bulgaria; AY508484, Turkey; AY366385, Iran; DQ076416, South Africa; DQ076415, Uganda; DQ227496, China; DQ217602, China; DQ227495, China; DQ214644, Russia; DQ133507 Kosovo; DQ211649, Turkey; U88412, Russia; U04958, Greece; U88410, Africa; U88415, Africa) were downloaded from the GeneBank and aligned using Clustal Wallis [9] software program. The sites of the possible primers were selected visually and evaluated using Primer test option of Primer Express software (Applied Biosystems^®^, USA).

Five forward and 2 reverse primers were designed initially. Based on the results of the optimization and assesment of the performance of the primers performed using CCHF virus cDNA and PCR optimization kit (Sigma^®^, Germany) with different Mg^++ ^and primer concentrations, two forward and one reverse primer were selected for routine use. All but one of the remaining primers were used as sequencing primers. The sequences of the primers numbered based on the sequence of Hodza strain (AY223475) (Table 1).

**Table 1:** The primers and nucleotide numbers according to the Hodhza strain

CCF-5F		aaa cac gtg ccg ctt acg cc	(5–24) (this study)
CCF-115F	First round	aar gga aat gga ctt rtg ga	(115–134) (this study)
CCF-131F	Second round	tgg aya cyt tca caa act cc	(131–152) *
CCF-295F	Sequencing	tgg gty agc tcy acy ggy att gt	(295–312) (this study)
CCF-457F	Sequencing	gac ata ggt tty cgt gty aat gc	(457–479) (this study)
CCF-479R	Sequencing	gca ttr aca cgr aar cct tat gtc	(479–457) (this study)

CCF-759R	First and second round	gca agg cct gtw gcr aca agt gc	(759–736) (this study)

* Modified from reference [23]

Five μl reverse transcription product was added to the 45 μl reaction mixture consisted of 1 μl forward primer (50 pmol/μl)) and 1 μl reverse primer (50 pmol/μl), 5 μl 10× reaction buffer, 3 μl 25 mM MgCl_2_, 1 μl dNTP stock (200 mM each dATP, dTTP, dCTP and dGTP) (Fermentas^®^, Lithuania), 0.25 μl Taq DNA polymerase (Fermentas^®^, Lithuania) and 33.75 μl nuclease free water. Amplification was carried out on a gradient thermal cycler under following conditions: After initial denaturation for 3 minutes at 94°C; 45 cycles of 1 minute at 94°C, 1 minute at 52°C and 3 minutes at 72°C. Final extention was performed at 72°C for 10 minutes. For the second round amplification 2 μl first round products was added to the 48 μl reaction mixture with the same concentrations of the first round mixture. Also the thermal cycling conditions were same. The amplification products were visualized on 1.5% agarose gel electrophoresis under UV-light.

The second round PCR products were cleaned up with a commercial PCR product purification kit (High Pure PCR Product Purification Kit, Roche Diagnostics^®^, Germany and the purified products were subjected to the cycle-sequencing using big-dye terminator kit (ABI^®^, USA). The excess primers and nucleotides were removed from cycle-sequencing products using sephadex-G50 fine columns, and the products were run on an automated sequencer (ABI^®^, 310).

The obtained sequences were edited and aligned using lasergene (DNA Star^®^) and Bioedit software packages [10]. The sequences belonging to five patients were deposited to the GeneBank under the accession numbers: DQ983741, DQ983742, DQ983743, DQ983744, DQ983745. For molecular analyses and comparisons, a portion spanning 593 (between 146–739. nucleotides; numbering according to Hodza strain, AY223475) nucleotides of the S segment was used. Phylogenetic analyses were carried out by distance method using neighbor joining algorithm with Treecon, version 1.3b software [11]. Distances were calculated under Kimura 2 parameter model. Transition/transversion ratio was estimated from the data. Neither insertions nor deletions were taken in to account. Topologic accuracy of the tree was evaluated by bootstrap method (1000 replicates) and only bootstrap values ≥65% were considered significant.

The informed consents from the patients were obtained. This was an observational study, which was conducted in compliance with Helsinki declaration.

## Results

### The characteristics of the cases

**Figure 2:**
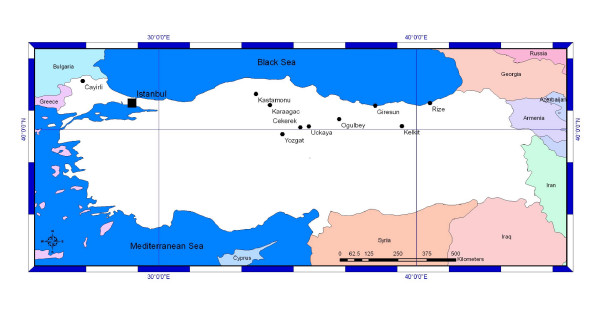
The Geographic Origin of the Imported Cases.

Between 12 June 2006 and the end of the October 2006, 91 CCHF suspected cases applied to the hospitals in the urban area of Istanbul (Figure 1). Among these, PCR bands corresponding to the expected size (628 bp) product of the S segment were detected in 10 patients. All PCR positive patients except one were also confirmed by IgM seropositivity. All of the PCR negative patients were also serologically negative. These patients were from Çorum (1), Giresun (1), Gümüşhane (1), Kastamonu (1), Kýrklareli (1), Rize (1), Tokat (2) and Yozgat (2) (Figure 2). Demographic, clinical and laboratory characteristics of the patients were depicted in Table 2 and Table 3.

**Table 2:** Demographic characteristics of the patients

	10 patients (%)
Number of females	6 (60)
Mean age (range)	40 (13–67)
History of tick bite	6 (60)
Days from symptoms to admission (median, min-max)	6.5 (3–7)
Length of stay in the hospital	10 (4–13)
Symptoms and signs	
Fever	10 (100)
Petechia	8 (80)
Maculopapular rash	6 (60)
Myalgia	8 (80)
Headache	8 (80)
Gingival bleeding	4 (40)
Vaginal bleeding	1 (10)
Epistaxis	5 (5)
Hematuria	1 (10)

Somnolence	0

**Table 3:** Pathologic laboratory findings of the patients

	10 patients (%) Median (min-max)
Longest Prothrombin time, s	11.6 (9.8–15.2)
Longest activated partial thromboplastin time, s	31.9 (26.6–40)
Lowest platelet count, platelets/mm^3^	50,000 (20,000–140,000)
Lowest WBC, WBCs/mm^3^	2900 (1800–3500)
Highest alanine transferase level, U/L	151 (53–319)
Highest aspartate transferase level, U/L	308 (47–581)
Highest lactic dehydrogenase level, U/L	940 (494–1657)

Highest creatinine phosphokinase level, U/L	266 (250–583)

### Sequence analyses

**Figure 3:**
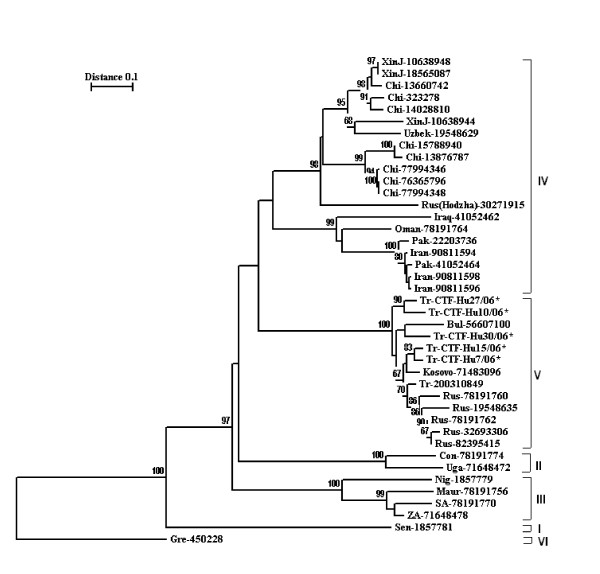
Phylogenetic tree for the CCHF strains. *: New Turkish strains detected by this study. I: West Africa. II: Democratic Republic of Congo. III: South/West Africa. IV: Asia/Middle East. V:Europe/Turkey. VI: Greece

Although the patients came from different localities of Turkey or had indirect contact with different endemic areas, the sequences of CCHFv were closely related. The nucleotide variation ranged between 0.66% and 3%, and this variation did not result in any amino acid substitution. In comparisons with the representative strains from different geographic regions, the strains detected in Istanbul showed highest homologies with Eastern European strains. Nucleotide and amino acid diversities varied between 1.16%–3.17% and 0–1 respectively (DQ1335071, DQ211643). Higher nucleotide and amino acid divergencies (21.20% and 20% respectively) were observed in comparisons with representative strains from Asian (AJ538196, M86625, DQ446212), and African (U88411, U88410, DQ076413) clades, and the Greek isolate (U04958). A total of 41 sequences of CCHFv S segment were used for the construction of the phylogenetic tree. In this tree the strains clustered with high bootstrap values reaching up to 100% in 6 clusters reflecting their different origins as shown previously. Our strains clustered in the Europe/Turkey clade [12] (Figure 3).

## Discussion

Since CCHF was endemic for the last four years in Eastern Anatolia, the *ad hoc *committee for CCHF in Istanbul initiated series of activities including the management of the suspected cases, in a city with the population of over 10 millions. A public awareness campaign was launched which included brochures, TV programs, and public health worker education programs about early diagnosis and preventive measures concerning tick bites and CCHF infections. As a result, there is improved surveillance and diagnosis of suspected cases (Figure 1). It is possible that previous introductions have gone unnoticed, especially since the majority of the cases appear to be relatively mild.

Suspected CCHF infections in people traveling from endemic regions to disease-free areas, such as Istanbul, were confirmed in 10 out of 91 cases. Nine out of 10 cases were from Eastern Anatolian provinces, where cases were reported previously [2,3,13]. However, one case originated from Kirklareli near the border with Bulgaria, which is the first report from this area (Figure 2). Imported cases of CCHF were reported previously, as cases from people traveling from one country to another [14,15].

Sixty percent of our patients reported the tick bite, and all the patiens had been bitten out of Istanbul (Table 2). The HCWs facing with the suspected cases should be aware that, a significant proportion of the suspected cases did not report tick bite, as it was previously published [1]. In South Africa, the time to onset of disease after exposure to tick bite was 3.2 days, to blood or tissue of livestock was 5 days, and to blood of human cases was 5.6 days [16]. The mean duration of the disease course before the hospital was reported to be 5.5 days in a previous study from Turkey [2]. However in our study, the average time from onset of disease to admission to hospital was 7 days (Table 2). None of the cases was fatal. The patients were given erythrocyte, fresh frozen plasma, and total blood preparations depending on their hemostasis. All the patients were given ribavirin therapy before the virologic evidence of CCHF infection.

Although the genetic heterogeneity of tick-borne RNA viruses is generally low, CCHFv shows a high level of genetic heterogeneity up to 22% nucleotide variations and 27% amino acid variations based on the S fragment, which is reflected into six genetically distinct clades [12]. Although confirmed patients came from 8 different localities in Turkey, all CCHFv sequences were closely related. The nucleotide variation ranged between 0.66% and 3%, and this variation did not result in any amino acid substitution. In comparison with representative strains from different geographic regions, those strains found in this study displayed the highest homology with the Eastern European and some Russian isolates (clade V)(Fig. 3). Also, CCHF is endemic in neighboring countries and genetically distant strains circulate in Bulgaria, Iran and Greece [17-19]. Moreover, in Iran, two different lineages of the virus have been reported; one belonging to the Asian-Middle Eastern clade and the other to the African clades [18]. Strains reported from Iraq and Arabian countries were closely related to those reported from Iran. Nevertheless, strains from the Eastern European countries and South-western Russia form a distinct group with relatively low heterogeneity (Figure 3).

Although relatively few sequences are available since the disease outbreaks were recorded in Turkey for the first time in 2003, those which are available are related to viral lineages found in Kosovo and South-western Russia [3]. Furthermore, viral strains in ticks collected from the endemic region where the first outbreaks occurred, were also found to be closely related with the Europe/Turkey strains [20,21]. In our study, all strains clustered within the same group with the Europe/Turkey isolates, despite they originated from eight different geographic areas, 7 previously known and one focus on the European part of the Turkey, which was identified in this study (Figure 3). Interestingly one strain (SPU 9/00/5) from Iran reported by Burt and Swaenopel [22] clustered also with Europe/Turkey clade.

Despite more viral strains are needed to draw any definite conclusion, it appears the emergence of CCHF in Turkey may be the result of an amplification of local viral isolates by wildlife populations rather than resulting from novel introductions from neighbouring countries. Continued surveillance to monitor suspected CCHF cases, not only within known foci, but also outside endemic areas as reported herein, is required.

## Competing interests

The author(s) declare that they have no competing interests.

## Authors' contributions

All authors read and approved the final manuscript

1. Kenan Midilli: study design, microbiological laboratory studies, analysis of the results and manuscript preparation

2. Ayşen Gargılı: study design, microbiological laboratory studies, analysis of the results and manuscript preparation

3. Onder Ergonul: study design, analysis of the results and manuscript preparation

4. Gönül Şengöz: study design, organization of the data collection, analysis of the results

5. Recep Ozturk: study design, analysis of the results

6. Mehmet Bakar: study design, organization of the data collection, analysis of the results

7. Frans Jongejan: manuscript preparation

## Pre-publication history

The pre-publication history for this paper can be accessed here:


